# Impact of nanoparticle surface functionalization on the protein corona and cellular adhesion, uptake and transport

**DOI:** 10.1186/s12951-018-0394-6

**Published:** 2018-09-15

**Authors:** Ashraf Abdelkhaliq, Meike van der Zande, Ans Punt, Richard Helsdingen, Sjef Boeren, Jacques J. M. Vervoort, Ivonne M. C. M. Rietjens, Hans Bouwmeester

**Affiliations:** 1 RIKILT-Wageningen Research, P.O. Box 230, 6700 AE Wageningen, The Netherlands; 2 Division of Toxicology, Wageningen University, P.O. box 8000, 6700 EA Wageningen, The Netherlands; 3 Food Science and Technology Department, Faculty of Agriculture, Alexandria University, Alexandria, Egypt; 4 Laboratory of Biochemistry, Wageningen University, P.O. box 8128, 6700 ET Wageningen, The Netherlands

**Keywords:** Nanoparticles, High throughput screening, Cellular adhesion and uptake, Label-free LC–MS/MS, Quantitative proteomics

## Abstract

**Background:**

Upon ingestion, nanoparticles can interact with the intestinal epithelial barrier potentially resulting in systemic uptake of nanoparticles. Nanoparticle properties have been described to influence the protein corona formation and subsequent cellular adhesion, uptake and transport. Here, we aimed to study the effects of nanoparticle size and surface chemistry on the protein corona formation and subsequent cellular adhesion, uptake and transport. Caco-2 intestinal cells, were exposed to negatively charged polystyrene nanoparticles (PSNPs) (50 and 200 nm), functionalized with sulfone or carboxyl groups, at nine nominal concentrations (15–250 μg/ml) for 10 up to 120 min. The protein coronas were analysed by LC–MS/MS.

**Results:**

Subtle differences in the protein composition of the two PSNPs with different surface chemistry were noted. High-content imaging analysis demonstrated that sulfone PSNPs were associated with the cells to a significantly higher extent than the other PSNPs. The apparent cellular adhesion and uptake of 200 nm PSNPs was not significantly increased compared to 50 nm PSNPs with the same surface charge and chemistry. Surface chemistry outweighs the impact of size on the observed PSNP cellular associations. Also transport of the sulfone PSNPs through the monolayer of cells was significantly higher than that of carboxyl PSNPs.

**Conclusions:**

The results suggest that the composition of the protein corona and the PSNP surface chemistry influences cellular adhesion, uptake and monolayer transport, which might be predictive of the intestinal transport potency of NPs.

**Electronic supplementary material:**

The online version of this article (10.1186/s12951-018-0394-6) contains supplementary material, which is available to authorized users.

## Background

Commercial, therapeutical and technological interests in engineered nanoparticles (NPs) are still increasing because of their unique physicochemical properties that make them promising materials for a wide range of new applications. NPs are currently being used in the agri-food sector in particular within domains like food processing, packaging, and as nutraceutical delivery systems [1–3]. The unique size-related properties may also pose a risk to human health because of their interactions with biomolecules, cells and organs, potentially leading to adverse outcomes [4, 5]. The oral route of exposure is considered one of the main exposure routes, especially for NPs exploited in agri-food applications. To assess the likelihood of NPs to internalize and cross the intestinal epithelial barrier several in vitro intestinal epithelial models have been developed [6–9]. Rapid screening of the intestinal transport potential of NPs is important in a tiered risk assessment or grouping approach [10].

The cellular uptake/transport of NPs is highly dependent on both the intrinsic and extrinsic properties of NPs. It is well known that intrinsic NP properties, such as size [6] and surface modifications affect cellular uptake [11, 12]. Upon contact with biological matrices like gastrointestinal juices and body fluids NPs are immediately covered with proteins generating the so-called protein corona [13]. The composition of the protein corona formed on the NPs surfaces is highly influenced by the physicochemical properties of the NPs [14, 15]. Consequently, the NP corona is considered as one of the major players affecting the biological interactions of the NPs, including their cytotoxicity, uptake and transport [14, 16, 17]. Additionally, the correlation between NP properties and their cellular uptake/transport appears to be cell type dependent, indicating that different kinds of uptake/transport mechanisms could be in place [18, 19]. Due to these complexities, no key descriptor has been identified so far for NP uptake/transport.

Here we aimed to study the effects of the size and surface chemistry of NPs on the protein corona formation and their subsequent cellular adhesion, uptake and transport. Several methods are available to study the cellular interactions of NPs at a single-cell level. High content (HC) imaging analysis has proven to be a highly successful and powerful tool in the field of drug discovery and toxicology, but it has rarely been used to study the behaviour and uptake of NPs [20, 21]. Here, HC imaging was used to study the cellular associations of fluorescently labelled, negatively charged, polystyrene nanoparticles (PSNPs) on a single-cell level, using Caco-2 monolayers as an in vitro method that mimics the human intestinal epithelium. Also, the transport of these PSNPs was assessed to gain insights into the correlation between the cellular adhesion/uptake and transport of these PSNPs. Lastly, the composition of the protein corona was quantitatively determined using label-free liquid chromatography mass spectrometry (LC–MS/MS).

## Methods

### Nanoparticles

Two 50 nm negatively charged, red fluorescently-labelled PSNPs with different surface modifications were obtained from Magsphere^®^ (Pasadena, USA). Namely; 2.5% w/v sulfonated particles and 2.5% carboxylated PSNPs, further referred to as 50 nm (-SM) and (-CM), respectively. 50 nm and 200 nm negatively charged, 2.5% w/v yellow-green fluorescently-labelled carboxylated PSNPs (Fluoresbrite^®^) were obtained from Polysciences (Warrington, USA), further referred to as 50 nm (-CP) and 200 nm (-CP). All PSNPs suspensions were stored at 4 °C and all experiments were performed using the same batch of PSNPs. Serial dilutions of the PSNPs were freshly prepared for every experiment in complete cell culture medium. Absence of detectable leakage of the fluorophores from the PSNPs used was confirmed by centrifugation of NPs after 24 h incubations in cell culture medium at 37 °C [9].

### Cell culture

Adherent human epithelial colorectal adenocarcinoma cells (Caco-2; ATCC^®^ HTB-37™), were used at passage numbers 25–40. They were cultured and maintained in 75 cm^2^ cell culture flasks (Corning^®^; New York, USA) at 37 °C in a humidified 5% CO_2_ atmosphere (HERAcell 240 incubator; Marietta, USA). Complete cell culture medium was prepared by supplementing Dulbecco’s Modified Eagle Medium (DMEM) culture medium (LONZA; Verviers, Belgium) with 10% (v/v) heat inactivated Foetal Bovine Serum (FBS) (Gibco^®^, Life technologies; New York, USA), 1% (v/v) of Penicillin–Streptomycin (Sigma-Aldrich; Steinheim, Germany), and 1% (v/v) of MEM Non-Essential Amino Acids (NEAA) (Gibco^®^, life technologies; New York, USA). The complete medium is further referred to as DMEM^+^.

### Physicochemical characterization of PSNPs

Size and surface charge of PSNPs were characterized using dynamic light scattering (DLS) and zeta-potential measurements, respectively. Briefly, DLS measurements were performed on 10 µg/ml PSNPs suspended in water and/or DMEM^+^ using an ALV dynamic light scattering setup (ALV-Laser Vertriebsgesellschaft; Germany), consisting of a Thorn RFIB263KF photomultiplier detector, ALV-SP/86 goniometer, ALV 50/100/200/400/600 μm pinhole detection system, ALV7002 external correlator, and a Cobolt Samba-300 DPSS laser. Each sample was measured 10 times for 30 s at an angle of 90°. The results are expressed as the hydrodynamic diameter that was calculated using AfterALV^®^ software (AfterALV 1.0d, Dullware; USA). The zeta-potential was measured using a Malvern Zetasizer 2000 (Malvern Instruments; Malvern, UK) on 10 µg/ml PSNPs suspended in DMEM^+^. All samples were analysed in triplicate.

### In vitro sedimentation, diffusion and dosimetry (ISDD) model for PSNPs

The deposited fraction of the administered doses of the PSNPs (target cell dose) was calculated using the In vitro Sedimentation, Diffusion and Dosimetry (ISDD) model [22]. The following parameters were used as input in the ISDD model: the hydrodynamic diameters of the PSNPs in water and DMEM^+^ measured by DLS (Table 1), medium column height (10.9 mm), temperature (310°K), media density 1 g/ml and media viscosity 0.0009 N s/m^2^ [23, 24].

**Table 1:** Physicochemical characteristics of PSNPs

PSNP	Hydrodynamic diameter (d_h_) (nm) of PSNPs in Water and DMEM^+^				Simulated fraction of nominal dose deposited (ISDD modelling)		Zeta-potential (mV) of PSNPs in DMEM^+^	
	Water (t = 0)	DMEM^+^ (t = 0 min)	DMEM^+^ (t = 30 min)	DMEM^+^ (t = 24 h)	DMEM^+^ (t = 30 min)	DMEM^+^ (t = 24 h)	DMEM^+^ (t = 0)	DMEM^+^ (t = 24 h)
50 nm (-SM)	52.9 ± 0.2	78.1 ± 11.6	83.3 ± 13.5	55.9 ± 9.6	0.011	0.092	− 13.3 ± 1.5	− 11.9 ± 0.9
50 nm (-CM)	43.9 ± 6.4	61.5 ± 12.4	58.3 ± 8.4	53.7 ± 20.4	0.013	0.093	− 10.2 ± 0.8	− 10.2 ± 1.1
50 nm (-CP)	52.8 ± 9	111.6 ± 23.1^a^	96.2 ± 20.8^a^	93.7 ± 19.2^a^	0.010	0.071	− 8.8 ± 1.5	− 9.2 ± 0.9
200 nm (-CP)	208.3 ± 7.4	267.3 ± 9.7^a^	238.4 ± 22.9	305 ± 9^abc^	0.007	0.045	− 11.1 ± 2.3	− 10.4 ± 0.6

Hydrodynamic diameters (nm) of PSNPs in water and DMEM^+^ (n = 3) and the zeta-potential (mV) in DMEM^+^ (n = 3)

(*-SM*) PSNPs functionalized with sulfone from Magsphere; (*-CM*) PSNPs functionalized with carboxyl from Magsphere, (*-CP*) PSNPs functionalized with carboxyl from Polysciences

^a^Significance difference versus water (0 min)

^b^Significance difference versus DMEM^+^ (0 min)

^c^Significance difference versus DMEM^+^ (30 min)

### Cell viability

Cytotoxic effects of the PSNPs were determined using a Cell Proliferation Reagent WST-1 (Roche; Mannheim, Germany). Each well was seeded with 1 × 10^5^ cells/cm^2^ in DMEM^+^ in 96-well flat bottom plates (Greiner bio-one; the Netherlands). Plates were incubated at 37 °C, 5% CO_2_ for 24 h. Attached cells were then exposed to 100 µl/well of freshly prepared serial dilutions of 50 nm- (-SM), (-CM), (-CP), and 200 nm (-CP) PSNPs (15, 25, 50, 75, 100, 200, 250, 500 and 750 µg/ml) for 3 and 24 h. Afterwards the exposure medium was discarded and 10 µl of WST-1 solution was added with 90 µl of DMEM^+^ (without phenol red) to each well. The plates were incubated for 24 h at 37 °C, 5% CO_2_ and absorbance was read at 490 nm and 630 nm on a plate reader (BioTek Synergy™ HT Multi-Mode Microplate reader; USA). Cell viability for each concentration of PSNPs was expressed as a percentage of the control. DMEM^+^ was used as a negative control and Triton-X100 (0.25%) (Sigma) was used as a positive control that decreased the viability to 29 ± 0.2%.

### PSNP cellular adhesion and uptake studies and HC imaging

A cell suspension of 5 × 10^4^ cells/cm^2^ was seeded in 96-well flat bottom black plates (Grenier bio-one; Frickenhausen, Germany) and incubated at 37 °C, 5% CO_2_ for 24 h. Subsequently, the culture medium was aspirated and cells were exposed to 100 µl/well of 15, 25, 50, 75, 100, 200 and 250 µg/ml of each of the PSNPs (n = 2) for 10, 20, 30, 60, and 120 min for the (-SM) PSNPs and for 30, 60, and 120 min for the other PSNPs. Exposure medium was then aspirated and the cells were washed once with 100 µl/well PBS buffer at 37 °C. As the PSNPs had different fluorescent labels, two different staining protocols were used. The cells exposed to the red 50 nm (-SM) and (-CM) PSNPs were incubated with a mixture (100 µl/well) of 4 µM Hoechst (Molecular Probes^®^, life technologies; USA) (blue; nucleus stain) and 1 µM Calcein AM cell permeant dye (Molecular Probes^®^, life technologies) (green; cytoplasm). The cells exposed to the yellow 50- and 200 nm (-CP) PSNPs were incubated with a mixture (100 µl/well) of 4 µM Hoechst (blue; nucleus) and 1 µM deep red MitoTracker (Molecular Probes^®^, life technologies) (red; mitochondria). Cells were incubated in the dark at 37 °C, 5% CO_2_ for 30 min.

Cellular adhesion and uptake of PSNPs was analysed using a Cellavista™ HC imaging system (SynenTec Bio Services; Munster, Germany). This HC imaging system uses an automated, quantitative fluorescence microscope with image acquisition and software to analyse multiparameter fluorescent cellular signals to quantify the local fluorescence intensity [25]. The output data were further processed using Microsoft Excel^®^2016 and Prism^®^ 5 software. PSNP adhesion and uptake was expressed as a fluorescence intensity per cell and as a median fluorescence intensity of the entire cell population. Distribution profiles of PSNP association and uptake in a cell population were made with GraphPad Prism^®^ 5. The number of cells correlating to each concentration bin was expressed as a percentage of the total number of exposed cells.

### PSNP cellular transport

Caco-2 cells were seeded at a density of 40,000 cells/cm^2^ on transwell permeable PET inserts (0.4 μm pore size, 1.12 cm^2^ surface area, Corning^®^; New York, USA). Cells were maintained for 21 days (37 °C, 5% CO_2_) and the apical and basolateral medium was changed every other day.

The integrity of the cell barrier was monitored by measuring the transepithelial electrical resistance (TEER) values with a chopstick electrode (STX01) connected to a Millicell ERS-2 Epithelial Volt- Ohm Meter (Millipore^®^; USA). Inserts with TEER values of 200 Ω cm^2^ and higher were used in the experiments. Additionally, the transport of lucifer yellow and 4- and 10 kDa- fluorescein isothiocyanate (FITC)-dextrans (Sigma-Aldrich; USA) was analysed by measuring the fluorescence intensity in the basolateral compartment after 1 h exposure at 37 °C at 485/530 nm using a BioTek Synergy™ HT Multi-Mode Microplate reader. Control samples received EGTA (Sigma-Aldrich) for 1 h at 37 °C to induce leakage of the cellular barrier (data not shown).

PSNPs exposure media were prepared at a concentration of 250 μg/ml in DMEM^+^, further diluted in DMEM^+^ when necessary, and directly applied apically onto the cells (500 μl/insert) on day 21 of culture. After 24 h of exposure, the basolateral medium was collected and fluorescence was measured at excitation/emission wavelengths of 530/590 nm and 485/530 nm, for red and yellow-green PSNPs, respectively using a microplate reader. The results are expressed as a percentage of transported PSNP from the total nominal dose of exposure. All experiments were conducted in triplicate.

### Confocal microscopy

For confocal microscopy, 1.5 × 10^4^ cells/cm^2^ were seeded into 8 well µ-Slides (Ibidi^®^; Martinsried, Germany) and incubated at 37 °C, 5% CO_2_ for 24 h. Afterwards, medium was discarded and cells were fixed with 200 µl/well of 4% paraformaldehyde for 15 min at room temperature. The fixation solution was discarded and cells were washed three times with 400 µl of PBS for 2–5 min, which was then replaced by 200 µl/well of permeabilization solution (FIX & PERM^®^ Cell Fixation & Cell Permeabilization Kit, life Technologies). After 15 min incubation at room temperature the cells were washed three times with 400 µl PBS for 2–5 min and incubated for 30 min at room temperature with 400 µl/well blocking buffer (1% BSA in PBS). The blocking buffer was discarded and 100 µl of LAMP-1 mouse primary antibody (a lysosomal marker) was added to the cells and incubated for 60 min at room temperature. Cells were washed three times with 100 µl PBS and 100 µl/well of the secondary antibody (Alexa Fluor 488 for cells exposed to the red PSNPs or Alexa Fluor 594 for cells exposed to the yellow PSNPs) added and incubated with the cells for 30 min at room temperature in the dark. The samples were then washed three times with PBS before addition of 100 µl/well of DAPI (Molecular Probes^®^, life technologies) which was incubated for 10 min at room temperature in the dark. Samples were washed three times with PBS and cells were stored in PBS 200 µl/well in the dark until analysis. The cells were analysed using a confocal laser scanning microscope (LSM 510-META, Zeiss, Germany) using 405, 488, and 543 nm lasers and the following filters for emission; BP420-480, BP505-530, and LP615.

### Characterization/quantification of protein corona of NPs

#### PSNPs protein corona collection

All 50 nm PSNPs at a concentration of 1 mg/ml and an equal total surface area of 200 nm PSNP were incubated in DMEM^+^ for 10, 20, 30, 60, 120 min at 37 °C. Afterwards, the samples were centrifuged (Hettich; Tuttlingen, Germany) for 40 min at 18,000*g*/15 °C. The pellets were three times re-suspended in 1 ml PBS and centrifuged for 25 min at 18,000*g*/4 °C. Laemmli loading buffer (Biorad—USA) containing β-mercaptoethanol was used to re-suspend the final pellet before boiling for 5 min at 95 °C followed by short centrifugation.

The total protein content in the samples was measured with a RC-DC Protein Assay (BIO-RAD) according to the manufacturer recommended protocol. All experiments were conducted in triplicate. DMEM^+^ was included as a control.

#### One dimensional sodium-dodecyl polyacrylamide gel-electrophoresis (1D - SDS-PAGE)

The required amount of protein (8 µg/well) was loaded onto pre-cast 12% SDS-PAGE gels of 1 mm thickness (BIO-RAD). 1D gel electrophoresis was then performed at 90 V for about 80 min. A protein ladder of 10–250 kDa was included in each gel. The gels were washed once with MQ water then with a water-based solution of 40% ethanol and 10% acetic acid for 15 min. Subsequently, the gels were stained overnight with Colloidal Coomassie Stain G-250 (BIO-RAD) on a rotating plate. After de-staining, the gels were scanned and the density of the bands was determined using an Odyssey scanner (Li-Cor ISO 9001, Odyssey Biosciences, Bad Homburg, Germany).

#### Proteomic analysis

On-beads digestion—as described in [26]—and µColumn (C18) cleaning procedures were applied to the protein corona samples before measurement by reversed-phase nano LC–MS/MS. Briefly, 18 μl of the collected protein corona samples of all PSNPs incubated in DMEM^+^ for 10, and 30 min at 37 °C were injected onto a Magic C18AQ 200A 5 µm beads (Bruker, USA) pre-concentration column (prepared in house) using a vacuum pump at a maximum pressure of 270 bar. Peptides were eluted and then injected into a 0.10 × 250 mm Magic C18AQ 200A 3 µm beads analytical column (prepared in-house) and eluted using an acetonitrile gradient at a flow of 0.5 μl/min with a Proxeon EASY nanoLC (Thermo Fisher Scientific, Waltham, MA, USA). The 1 h gradient consisted of an increase from 8 to 33% acetonitrile in water with 5 ml/l acetic acid in 50 min, followed by a fast increase up to 80% acetonitrile in water and 5 ml/l acetic acid (in both the acetonitrile and the water) in 3 min as a column cleaning step. Following, an electrospray potential of 3.5 kV was applied.

Full scan positive mode fourier transform mass analysers (FTMS) spectra were measured between m/z 380 and 1400 on a LTQ-Orbitrap XL (Thermo electron, San Jose, CA, USA) at high resolution (60,000). Tandem mass spectrometry (MS/MS) scans of the four most abundant 2 and 3 + charged peaks in the FTMS scan were recorded in a data dependent mode in the linear trap. LCMS runs with all MS/MS spectra obtained were analysed with MaxQuant 1.5.2.8 [27, 28].

The concentrations of the identified proteins from the PSNP coronas were determined using MassPREP tryptically digested standards (Water; Milford, USA). Standards contained a mixture of yeast enolase (SwissProt P00924), phosphorylase b (SwissProt P00489), bovine haemoglobin (SwissProt HBA P01966, HBB P02081), yeast alcohol dehydrogenase (ADH, SwissProt P00330) and bovine serum albumin (BSA, SwissProt P02769) dissolved into a range of concentrations between 0.5 and 8 pmol in 1 ml/l formic acid. Before analysis on the LC–MS/MS, 5 µl of each concentration of each standard was mixed with a sample of all NPs. This sample consisted of protein coronas isolated from equal amounts of all PSNPs used in this study after the digestion and cleaning up procedures—in a final volume of 50 µl in 1 ml/l formic acid.

#### Data processing and analysis

To quantitatively identify the proteins in the PSNP coronas a bovine database downloaded from Uniprot (release July 2016) (http://www.uniprot.org, 20.343 entries) [29], as well as a small database containing the four internal standard proteins were used together with a contaminants database that contains sequences of common contaminants like Trypsins (P00760, bovine and P00761, porcine) and human keratins (Keratin K22E (P35908), Keratin K1C9 (P35527), Keratin K2C1 (P04264) and Keratin K1CI (P35527)) [26]. The “label-free quantification” options were enabled and the MaxQuant protein Groups output file was filtered stringently by accepting only peptides and proteins with a false discovery rate (FDR) of less than 1% and proteins with at least two identified peptides of which at least one should be unique and at least one should be unmodified.

From the set of proteins standards, iBAQ intensities of phosphorylase b (SwissProt P00489) were selected for quantification of all proteins identified in the PSNP corona and DMEM^+^ samples. Identified proteins were grouped based on their biological function using the aforementioned Uniprot database [14]. The mass of each group was expressed as a percentage of the total mass of proteins and as number of molecules per cm^2^ (total copy number).

### Statistical analysis

Each data point represents the average of three independent experiments (n = 3) and the results are shown as a mean ± standard deviation after analysis by Prism^®^ (v.5.0; GraphPad^®^, USA) software. A one-way analysis of variance (ANOVA) with a Tukey’s post-test was used to test statistical significance after testing the normality distribution of the data sets using Kolmogorov–Smirnov test. A *P*-*value* < 0.05 was considered significant.

## Results

### Physicochemical characterization of the PSNPs

To characterize and assess the stability of the PSNP suspensions, the hydrodynamic diameters (d_h_) and zeta-potentials (ζ-potential) were measured in DMEM^+^ at the same incubation times used in the experiments. Compared to the samples in water among all PSNPs tested, only the 50 and 200 nm (-CP) showed a significant increase in size upon incubation in DMEM^+^, while the incubation time showed a significant influence only on the size of 200 nm (-CP). The ζ-potentials of all the PSNPs suspended in DMEM^+^ were similar and stable during 24 h incubation (Table 1).

### Cell viability

**Fig. 1:**
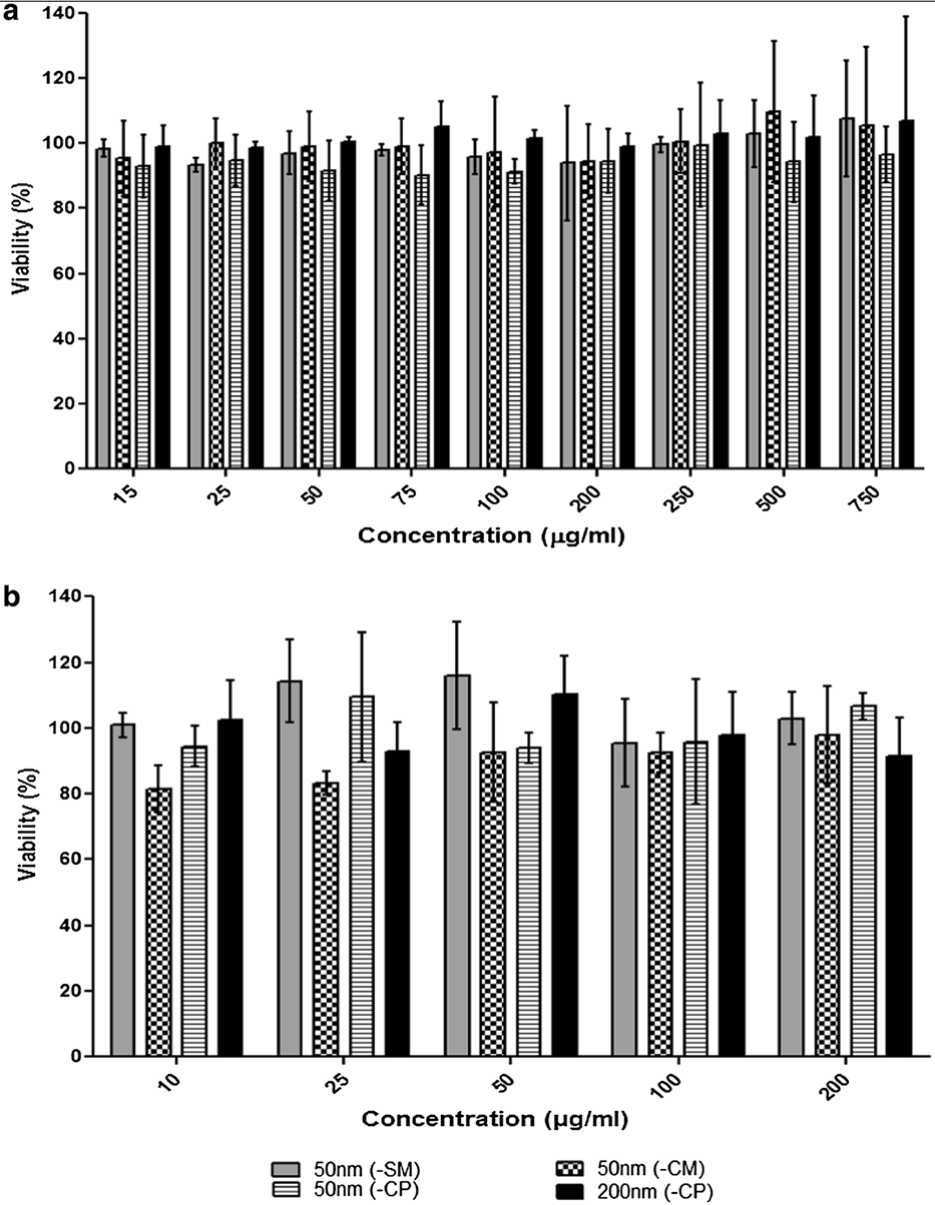
Caco-2 cell viability after exposure to a concentration series of 50 nm- (-SM), (-CM), (-CP), and 200 nm (-CP) PSNPs for **a** 3 h and **b** 24 h using the WST-1 viability assay. Viability is given as a percentage of the control (% ± SD; n = 3)

Cytotoxicity experiments (WST-1 assay) were performed to derive non-toxic concentrations of PSNPs for the uptake studies. Results demonstrated that after 3 h and 24 h exposure the cellular viability of Caco-2 cells was not affected (viability was always higher than 85% and 80% after 3 h and 24 h, respectively, compared to controls) in any of the concentrations tested (Fig. 1a, b).

### Cellular adhesion and uptake of PSNPs

**Fig. 2:**
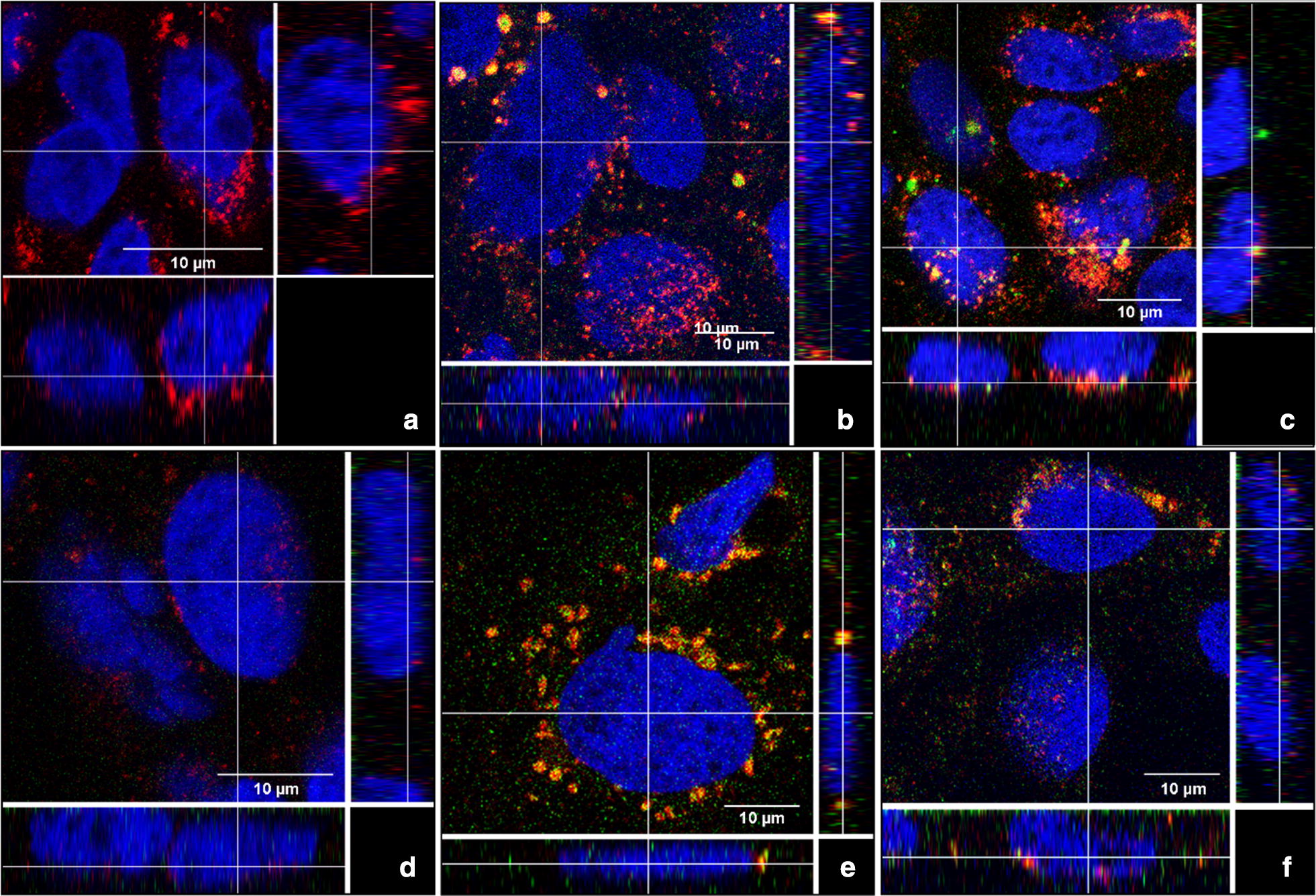
Confocal microscopy images of Caco-2 cells **a** w/o exposure to PSNPs—as control for the (-SM) and (–CM) PSNPs. After exposure for 24 h to a nominal concentration of 25 µg/ml **b** 50 nm (-SM) and **c** 50 nm (-CM). **d** Caco-2 cells—w/o exposure to PSNPs—as control for the (-CP) PSNPs. After exposure for 24 h to **e** 50 nm (-CP) and **f** 200 nm (-CP). Nuclei were stained in blue, lysosomes in red and PSNPs in green

Cellular association of PSNPs was quantified using HC imaging analysis. The HC images are taken from above the cells, thereby merging the fluorescent signal of both internalized and membrane adhered PSNPs into one image. PSNP internalization in Caco-2 cells was therefore confirmed using confocal microscopy (Fig. 2). The PSNPs partially co-localized with lysosomes after 24 h exposure indicating (partial) internalization of the PSNPs in the lysosomes.

**Fig. 3:**
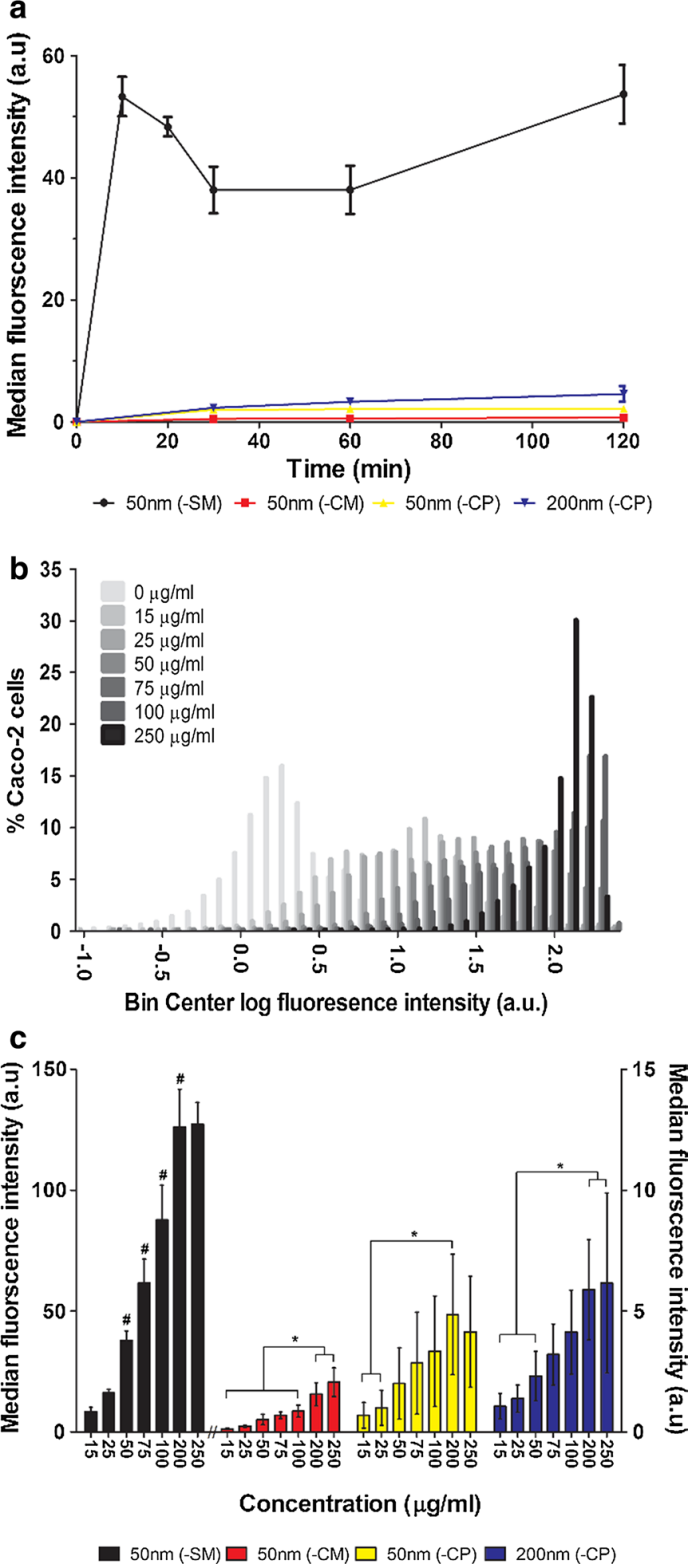
Cellular association of PSNPs with a different surface chemistry by Caco-2 cells. Adhesion and uptake was determined by single-cell HC image analysis of PSNP fluorescence. **a** Time dependent adhesion and uptake of four types of PSNPs in Caco-2 cells exposed to a nominal concentration of 50 µg PSNPs/ml for 10 up to 120 min. **b** Cellular association distribution profiles of 50 nm (-SM) PSNPs in the entire cell population at exposure to nominal concentration ranging from 15 to 250 µg/ml for 30 min. Note that the readings at the highest concentrations are hampered by saturation of the HC signal. **c** Concentration dependent cellular association of four types of PSNPs after 30 min of exposure (//; the fluorescence intensities of 50 nm (-SM) is plotted on the left y-axis while the rest of the PSNPs fluorescence intensities are plotted on the right y-axis). ^#^Significant difference versus all lower concentrations (P < 0.05). *Significant difference between indicated concentrations (P < 0.05)

PSNP adhesion and cellular uptake was measured on a cell-per-cell basis using an HC imaging system during 10–120 min exposure to concentrations ranging between 15 and 250 µg/ml and expressed as median fluorescence intensity per cell. The cellular adhesion and uptake of the 50 nm (-SM) PSNPs by Caco-2 cells was, at all time-points, significantly higher compared to the 50 nm (-CM), (-CP) and 200 nm (-CP) PSNPs (Fig. 3a). Cellular distribution profiles show that cellular association with the PSNPs increased upon increasing the PSNP concentration, seen as a right shift of the median in the adhesion and uptake distribution curves (Fig. 3b). The graph also shows that at concentrations of 75 µg/ml and higher part of cell population have fluorescence signals that reached or exceeded the maximum detection limit of the HC imaging system. For all PSNPs cellular adhesion and uptake increased with increasing concentration (Fig. 3c). At the higher concentrations the increase of cellular adhesion/uptake declines and stops, which is most likely due to the detection limit of the system. For the PSNPs that associated to the largest extent with the cells, namely the 50 nm (-SM) PSNPs, the cellular adhesion and uptake distribution profile over the entire cell population was further analysed (Fig. 3b) at the single cell level with is possible with HC imaging. The results obtained clearly point at an increased fluorescent signal associated with single cells with increasing dose levels. Finally, the apparent cellular adhesion and uptake of the two PSNPs with different sizes (i.e. 50 and 200 nm) but with the same surface chemistry, was not significantly different at all concentrations and time points tested (Fig. 3c). However, the ISDD model output (Table 1) that indicates a 1.4 fold lower fraction deposited of the 200 nm (-CP) PSNPs at 30 min compared with the 50 nm PSNPs with the same surface chemistry. The apparent cellular adhesion and uptake is comparable for both PSNPs. After correction of the lower deposition of the 200 nm (-CP) PSNPs the cellular adhesion and uptake of the 200 nm (-CP) PSNPs at 30 min is 1.4 fold higher for the 200 nm (-CP) compared with the 50 nm (-CP) PSNPs with the same surface chemistry.

### Transport of PSNPs across a monolayer of Caco-2 cells

**Fig. 4:**
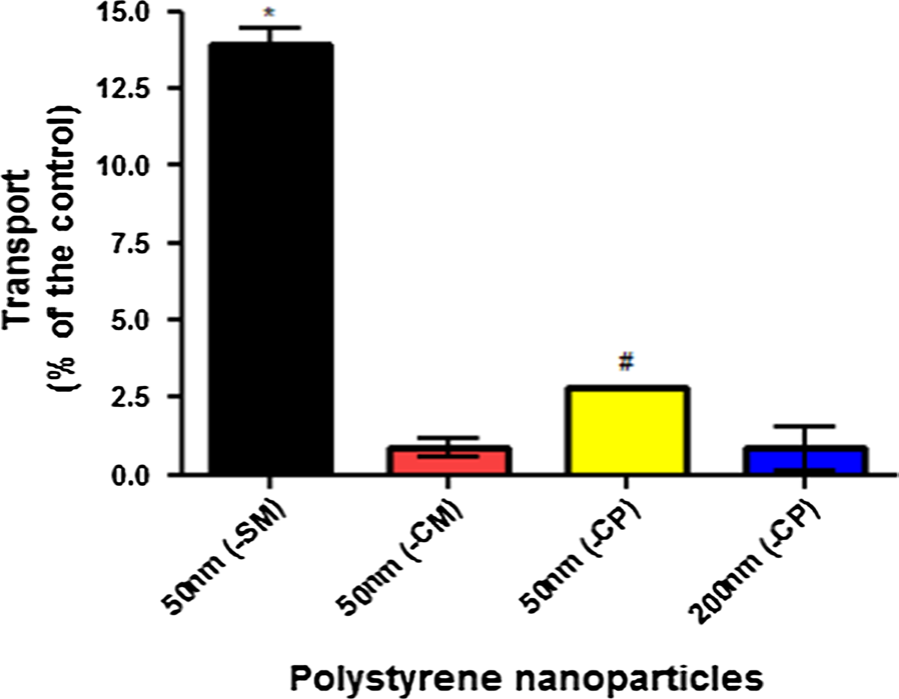
Transport of PSNPs with a different surface chemistry by Caco-2 cells. Transport of 4 types of PSNPs in Caco-2 cells exposed to a nominal concentration of 250 µg PSNPs/ml for 24 h. *Significant difference from all and ^#^significant difference from 50 nm (-CM) and 200 nm (-CP) PSNPs (P < 0.05)

To assess if cellular adhesion and uptake of PSNPs is predictive for transport across a monolayer of Caco-2 cells we performed additional experiments. A differentiated monolayer of Caco-2 cells was exposed to 250 µg/ml of each of the four PSNPs for 24 h. The longer exposure of 24 h was required to reach detectable concentration in the apical compartment of the transwell system. The transport markers used including the lucifer yellow and dextrans showed very minimal transport through Caco-2 monolayers and upon the application of EGTA to open the tight junctions, the permeability of these monolayers increased significantly (data not shown), indicating the functionality of tight junction integrity of the monolayers used in the transport studies [9, 30]. Transport of the PSNPs was determined by PSNP fluorescence measurement in the basolateral medium post exposure and expressed as a percentage of the amount of PSNP fluorescence in the medium that was applied apically at the start of the experiment (Fig. 4). The 50 nm (-SM) PSNPs showed the highest transport among all PSNPs tested (13.9%; p < 0.05), followed by the 50 nm (-CP) PSNPs (2.82%; p < 0.05). While the transport of the other carboxylated PSNPs—50 nm (-CM) and 200 nm (-CP)—did not show significant transport to the basolateral compartment (< 1%). However, if the 1.6 fold lower deposited PSNPs fraction after 24 h incubation of the PSNPs is taken into account (Table 1), the amount of the 200 nm (-CP) PSNPs is comparable to the transported amount of the 50 nm PSNPs with the same surface chemistry. Here we assume that the transport of the PSNPs increases with the concentration at the cell surface as shown for the cellular adhesion and uptake (Fig. 3).

### Characterization of PSNPs protein corona

**Fig. 5:**
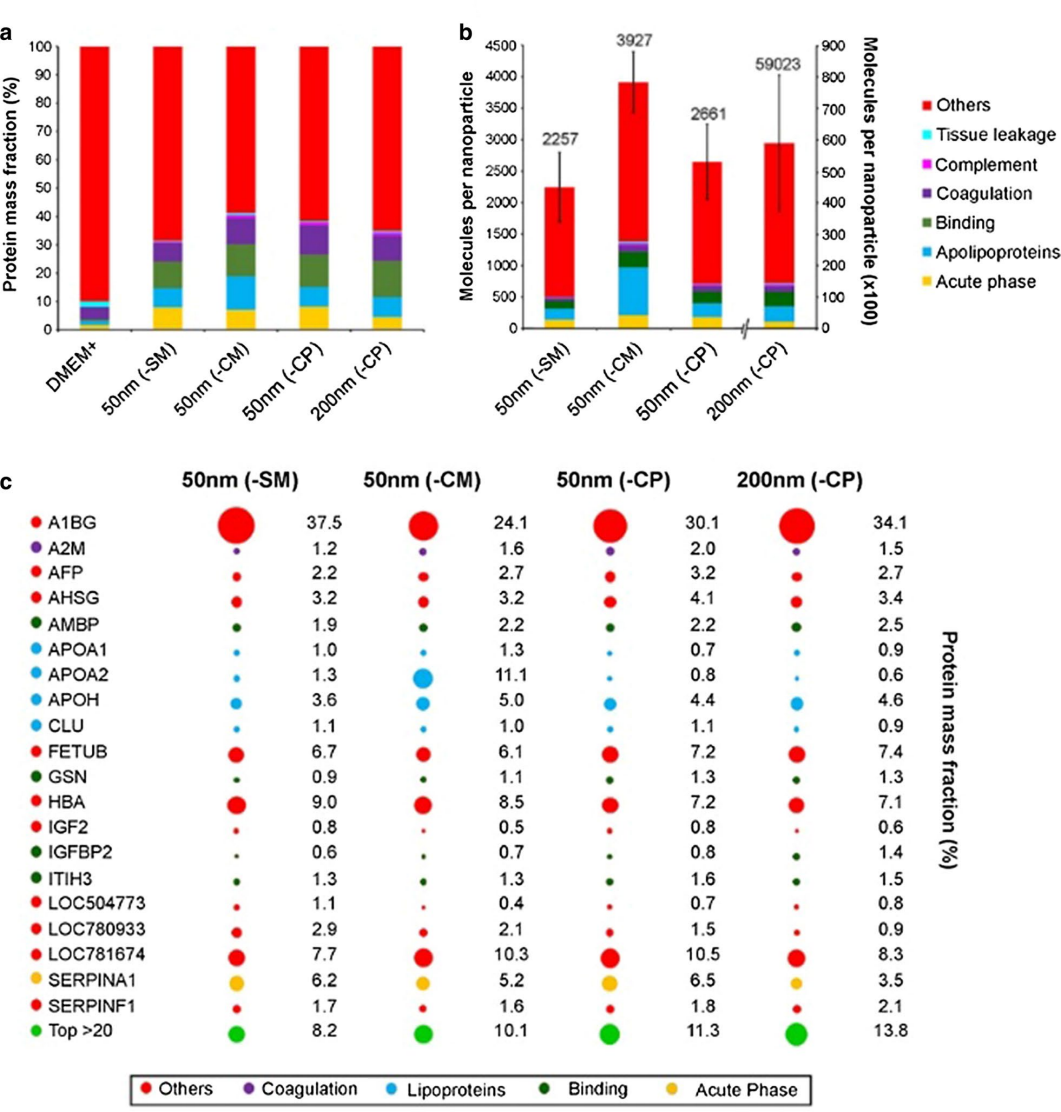
Composition of the protein corona on PSNPs with a different surface chemistry determined with LC–MS/MS. The proteins were classified into seven groups according to their biological function using proteomics databases. **a** Distribution of the protein groups in DMEM^+^ and in the protein coronas of the PSNPs, expressed as a percentage of the total protein mass. **b** Number of protein molecules per particle clustered per protein group. **c** Top 20 proteins with the highest protein adsorption on the respective PSNP. Proteins are ordered alphabetically. The colour code indicates the protein group and the size of the spot represents the mass fraction (%), which is also given in numbers

**Fig. 6:**
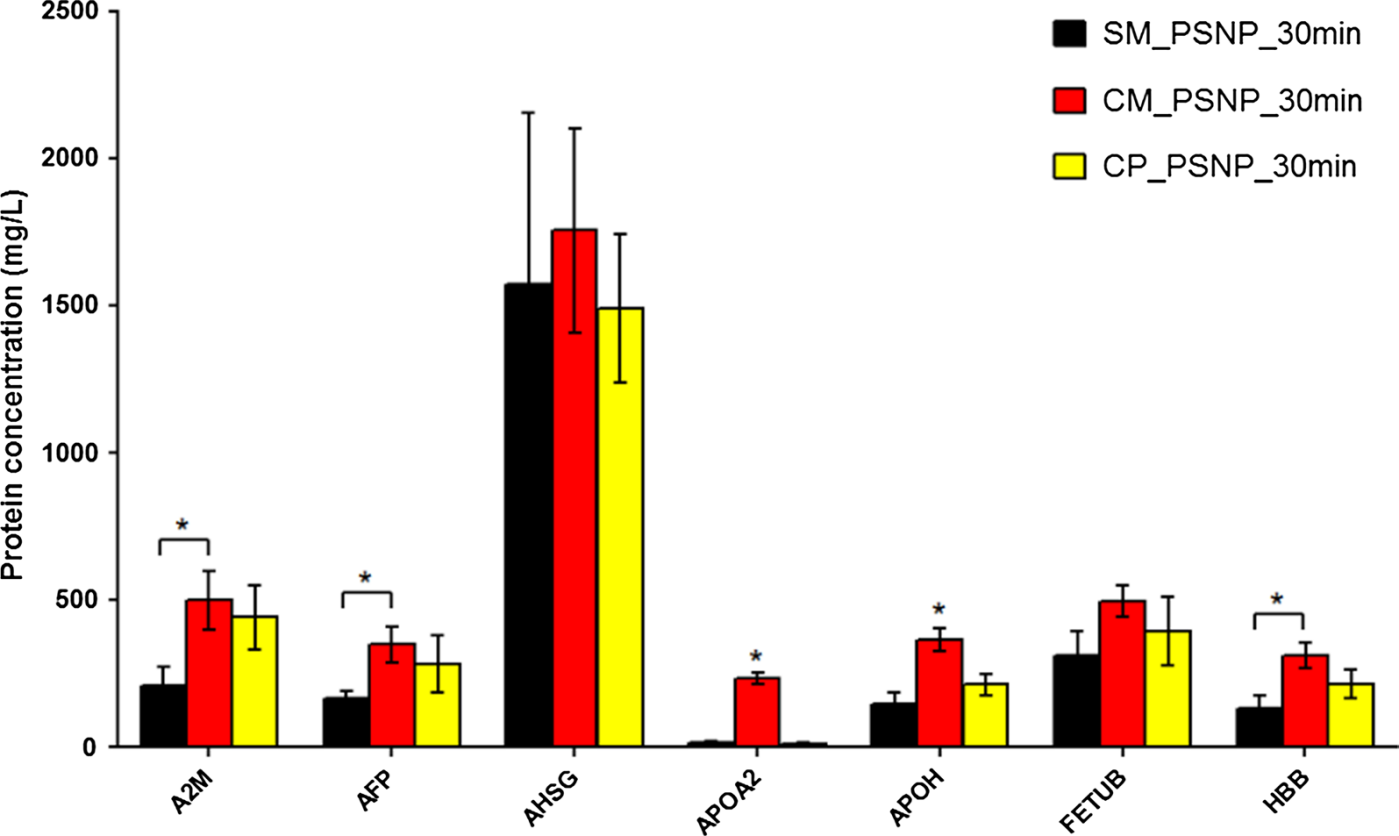
Comparison of differently adsorbed proteins to the surface of 50 nm PSNPs with a different surface chemistry. Only proteins with 2 or more copy numbers on at least 1 of the 3 different PSNPs were considered. Asterisks indicate significance difference (P < 0.05)

All PSNPs were incubated in DMEM^+^ at 37 °C for 10 and 30 min. The protein corona was then collected and analysed using SDS-PAGE. The gel was loaded with the same amount of protein for all tested PSNPs. The gels did not show large differences between the different PSNPs nor between the 10 and 30 min incubation time (Additional file 1: Figure S1). Furthermore, the protein corona was quantified and characterized using label-free nano-LC/MS–MS, which resulted in approximately 172 different adsorbed proteins. The complete list of identified proteins is provided in Additional file 2: Table S1. Proteins were clustered into 6 classes according to their biological function (and 1 “other” group). Comparing the relative amount (i.e. abundance) of a protein class on each nanoparticle to the protein class distribution as present in the cell culture medium, we observed an enrichment of proteins involved in binding, apolipoproteins, and acute phase proteins and a less pronounced enrichment of coagulation and complement factors (Fig. 5a). Subsequently, the total number of protein molecules per particle was calculated. On a single 50 nm PSNP around 2200–4000 protein molecules were absorbed (with the number of proteins on the different types of 50 nm PSNPs not being significantly different), while on the 200 nm PSNP about 59,000 proteins were absorbed (Fig. 5b). The number of proteins per surface area is between 3 × 10^13^ and 5 × 10^13^ proteins per cm^2^ for the 50 nm while it is about 6 × 10^13^ proteins per cm^2^ for the 200 nm PSNPs. Comparable or slightly more proteins are absorbed per cm^2^ on the 200 nm sized PSNP compared to the 50 nm PSNPs, an observation that might be explained by less steric hindrance on the larger particles. Among the top 20 most abundant proteins in the coronas of the 4 PSNPs, the protein Alpha-1B-glycoprotein (A1BG) ranked highest (Fig. 5c). More detailed statistical evaluation of the protein concentration on a single protein level in the coronas of the three different 50 nm PSNPs demonstrated differences in Alpha-2-macroglobulin (A2M), Alpha-fetoprotein (AFP), Apolipoprotein A-II (APOA2), Beta-2-glycoprotein 1 (APOH), and Hemoglobin fetal subunit beta (LOC781674 or HBB). Corona concentrations of most of these proteins we lower in the corona’s of the 50 nm (-SM) PSNPs, this was reaching significance only compared to the (-CM) PSNPs for the A2 M, AFP and HBB, while compared to both (-CM) and (-CP), the concentrations proteins APOA2 and APOH were lower in the corona’s of (-SM) (Fig. 6). For this evaluation only proteins with two or more copy numbers on at least one of the three different PSNPs were considered (~ 70 proteins in total).

## Discussion

Here, the effects of nanoparticle (NP) size and surface chemistry on the protein corona formation and subsequent cellular association (i.e. adhesions and uptake) and transport across a monolayer of Caco-2 cells is reported. Fluorescently labelled PSNPs were selected as model particles owing to their dispersion stability in cell culture media and the commercial availability in different sizes and surface modifications.

Characterization of the PSNPs, by determining the hydrodynamic size and zeta-potential of the PSNPs in cell culture medium, showed that all 50 nm PSNP suspensions were stable during the exposure times in the experiments. The DLS method as used here has been evaluated in a inter laboratory testing project [31] to characterize the size of the PSNPs in water or DMEM + and it was found that the proteins present in the cell culture medium resulted in a signal indicating a hydrodynamic diameter of 27 ± 2 nm. The zeta-potential of all PSNPs in cell culture medium was comparable and stable over time, irrespective of the PSNP surface chemistry with a negative charge on the PSNPs’ surface results from the PSNPs, the proteins adsorbed on the surface of the PSNPs or from measuring protein aggregates of medium rather than PSNPs [9].

The absence of cytotoxic effects of all PSNP concentrations used in this study was in concordance with findings from previous studies using 50 nm PSNPs functionalized with carboxyl (-CM and -CP) and sulfone (-SM) groups, using a variety of cell models [21, 32]. Confocal microscopy imaging in different planes of the cells showed internalization of the PSNPs, and partial co-localization of the PSNPs with lysosomes after 24 h of exposure. Thereby, confirming previous studies indicating uptake of negatively charged 60 nm and 100 nm PSNPs [33], and 50 nm and 100 nm silica (SiO_2_) NPs into the lysosomes [34, 35] and localization of NPs in the cytoplasm around the nucleus (for 50 nm, 90 nm, and 100 nm PSNPs) [35–38].

The cellular fluorescence as determined by HC resulting from sulfone (-SM) functionalized PSNP associated with Caco-2 cells was significantly higher compared to cells exposed to carboxylated (-CP and -CM) PSNPs of the same size. Cellular association of all functionalized PSNPs was dose dependent. Contrary to our expectations, no linear phase in the PSNP uptake kinetics by Caco-2 cells was observed, or this happened before the first time point (10 min) assessed in this experiment. Accordingly, no uptake rates of these PSNPs could be derived. The PSNPs association kinetics as observed here could be due to two processes taking place in parallel; namely PSNP cell membrane adhesion and cellular internalization. Lesniak et al. has reported very comparable time dependent changes in cellular fluorescence intensities in A549 (carcinoma human alveolar basal epithelial cells) exposed to 40 nm PSNPs over time. It was shown that during the first 10 min of exposure, cellular adhesion of PSNPs occurs rapidly, whereas after that the adsorption grows much slower [34, 39, 40]. This confirms the fast increment in fluorescence at the cells as observed by us, in the first 10 min of exposure. Recent modelling of these processes (in A549 cells) confirmed these time lines, until 20 min after exposure the PSNPs were mainly associated with the cell membranes, while after 2–3 h exposure, the PSNPs were found close to the lysosomes located centrally in the cells [33]. The subsequent increment in fluorescence between 60 and 120 min as we reported here, might suggest PSNP uptake by the Caco-2 cells. In addition, earlier studies using 100 nm polylactic polyglycolic acid (PLGA) NPs showed that their uptake by Caco-2 cells is taking place between 1 and 2 h of exposure [41].

Earlier studies focused on potential optimal size for cellular uptake. By exposing Caco-2 cells to 25, 50, 100, 200 and 500 nm PSNPs preferred uptake of 100 nm PSNPs was found using a microplate reader [42]. Uptake of 50 nm mesoporous silica NPs by HeLa cells was higher compared to 100 nm NPs after 5 h exposure [43]. However, in EAhy926 cells, uptake of 200 nm PSNPs was higher than 20 nm PSNPs after 24 h exposure to [44]. Comparing our observations and the data from literature point to cell type specific differences in uptake processes [14, 45]. Here we only compared studies using NPs with a comparable (effective) density. Comparing NP uptake data between studies needs to be performed with care as the reported cellular uptake is largely dependent on the particokinetics (i.e. diffusion and sedimentation) that is affected by the effective density of NPs [22]. Indeed when correcting the applied concentrations for the ISDD estimated deposited fraction [22], the cellular association/uptake of 200 nm (-CP) PSNPs becomes up to 1.4 fold higher compared to 50 nm PSNPs with the same surface chemistry, whereas without this correction the estimated uptake levels were found to be similar. Nonetheless, the surface chemistry was observed to outweigh the impact of size on the observed PSNP cellular associations with the sulfone functionalized PNPSs being higher associated to the cells than the carbonyl functionalized nanoparticles. Reported differences in NP uptake thus not always reflect differences in biological processes, but merely the physiochemical interaction of NPs with the exposure media.

The type of surface functionalization on NPs was found earlier to be one of the major factors determining adhesions, uptake, transport, and distribution of NPs, which appears to be mainly driven by the size of the protein corona [9, 46]. Here, we extended our previous semi-quantitative protein corona analysis using SDS-PAGE (Additional file 1: Figure S1) with a quantitative proteomic analysis using label-free LC–MS/MS, as described by [14]. As previously, and commonly, done, the present study was performed in the presence of fetal bovine serum. It should be noted that different types of protein mixtures are being used for in vitro studies, which might affect the outcome and thus the comparability of studies [45]. The proteomic analysis showed that the protein corona isolated from the PSNPs was composed of 172 different proteins. We observed an enrichment of proteins involved in binding, apolipoproteins, and acute phase proteins’ groups and a less pronounced enrichment of complement factors proteins group. Among these proteins, the alpha-1B-glycoprotein (A1BG) protein was most abundantly present on all types of NPs, but the function of this protein is currently unknown. In other studies apolipoproteins have been identified as a dominant protein group in NP protein coronas [47–49]. Comparing the protein corona composition among the PSNPs studied here, it was found that A2M, AFP, APOA2, APOH and HBB proteins were significantly less absorbed on the (-SM) functionalized PSNPs Strikingly, the cellular association and transport across a monolayer of Caco-2 cells of these (-SM) functionalized PSNPs was also significantly different compared to the carboxylated PSNPs. Two studies have successfully attempted to correlate the composition of the protein composition of the nanoparticle corona with cellular interaction [50, 51]. In these two correlative studies different proteins were identified to correlate with cellular association of NPs. Some of the identified apolipoproteins and A2M were also found in our study to be differently enriched in the coronas of our PSNPs. It has however proven to be difficult to identify a set of specific proteins that is directly linked (mechanistically) with cellular membrane association and subsequent NP update. Previously, using corona enrichment studies, it has been described that APOA2 and APOH interfere with the cellular uptake of NPs [14]. Here we show that different surface chemistry of PSNPs to some extent specifically enriches some proteins, like lipoproteins, binding proteins, and acute phase proteins in the corona. Some of these proteins have been associated with differential cellular interaction. A yet unresolved question is, what effects the presence of exogenous proteins from for instance food (allergy epitopes) proteins can have on the cellular interaction, as it was found that specific food related proteins in the NP corona enhance the uptake of these NPs [52].

Cellular association experiments using 24 h old Caco-2 cells can be performed much faster compared to monolayer transport studies that require 21 days to differentiate Caco-2 cells. Therefore, cellular association profiles of the PSNPs studied here were compared with transported amounts of these PSNPs across a monolayer of Caco-2 cells. Both, the cellular association of the (-SM) functionalized PSNPs and transported amount of these particles were higher than for the other PSNPs. This supports the previous conclusion that the adhesion properties of NPs to the cell membrane are key determinants of NP uptake [40], and thus likely also predictive for NP transport and could serve as a rapid screening of the intestinal transport potential of NPs is important in a tiered risk assessment or grouping approach [10].

## Conclusions

The cellular uptake of NPs is highly dependent on both the intrinsic and extrinsic properties of NPs. Based on our findings, we conclude that NP surface functionalization is a more important NP property than NP size, for the cellular association of PSNPs. The type and composition of the protein corona formed on the NP surface is affected by the physicochemical properties of the NP. The protein corona is consequently one of the major players affecting the NP cellular interactions including their cytotoxicity, membrane adhesion, uptake and transport. Further studies are required to identify the set of corona proteins that affect the uptake and transport of NPs. Membrane adhesion and cellular uptake profiles correlate with the observed transport across a monolayer of Caco-2 cells, indicating membrane adhesion studies can potentially be used to predict the transport potential of NPs.

## Additional files

**Additional file 1: Figure S1.** SDS-PAGE showing the protein corona of PSNPs.

**Additional file 2: Table S1.** The list of identified PSNPs corona proteins using LC-MS/MS after 10 and/or 30 min incubation in DMEM+.
