# Supplementary material for: Impact of nanoparticle surface functionalization on the protein corona and cellular adhesion, uptake and transport
Source: J Nanobiotechnology. 2018 Sep 15;16:70. doi: 10.1186/s12951-018-0394-6 (PMC6138932; doi:10.1186/s12951-018-0394-6)
Supplement: Supplementary file 1 — Additional file 1: Figure S1. SDS-PAGE showing the protein corona of PSNPs. [file 12951_2018_394_MOESM1_ESM.docx]

# Supplemental online material

**Impact of Nanoparticle Surface Functionalization on the Protein Corona and Cellular Uptake and Transport**

*Ashraf Abdelkhaliq^1,2,3^, Meike van der Zande^2^, Ans Punt^2^, Richard Helsdinger^2^, Sjef A. Boeren^4^, Jacques J.M. Vervoort^4^ and Hans Bouwmeester^1,2 *^*

^1^ Division of Toxicology, Wageningen University, P.O. box 8000, 6700 EA, Wageningen , the Netherlands

^2^ RIKILT - Wageningen Research, P.O. Box 230, 6700 AE, Wageningen, the Netherlands

^3^ Food Science and Technology Department, Faculty of Agriculture – Alexandria University,
 Alexandria, Egypt

^4^ Laboratory of Biochemistry - Wageningen University, P.O. box 8128, 6700 ET, Wageningen , the Netherlands

^*^ Corresponding author: email: [hans.bouwmeester@wur.nl](mailto:meike.vanderzande@wur.nl)

**Keywords:** Nanoparticles, High throughput screening, cellular uptake, label-free LC/MS, quantitative proteomics

C)

F

A)

B)

F

D)

F


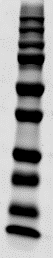

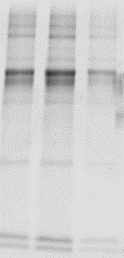

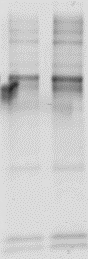

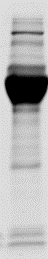


250

150

100

75

50

37

25

20

15

10

10min

20min

30min

60min

120min

DMEM^+^


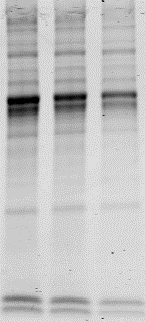

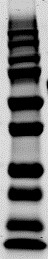

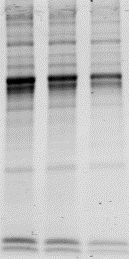

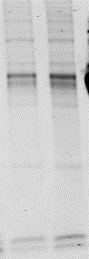

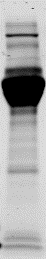


10min

20min

30min

60min

120min

DMEM^+^

250

150

100

75

50

37

25

20

15

10

10 min

20 min

30 min

60 min

120 min

DMEM^+^+

250

150

100

75

50

37

25

20

15

10


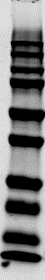

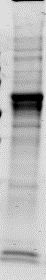

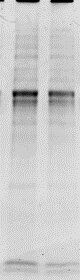

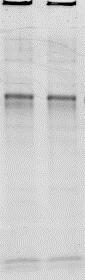

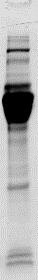


10min

20min

30min

60min

120min

DMEM^+^


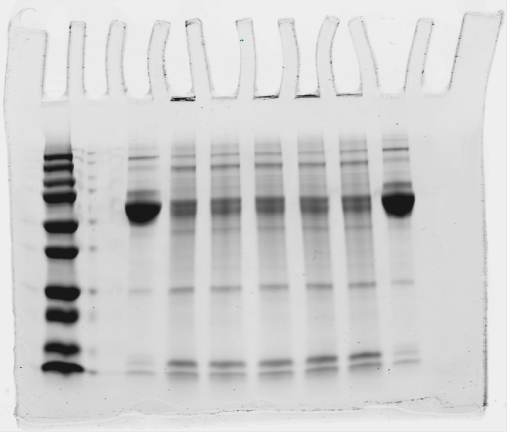

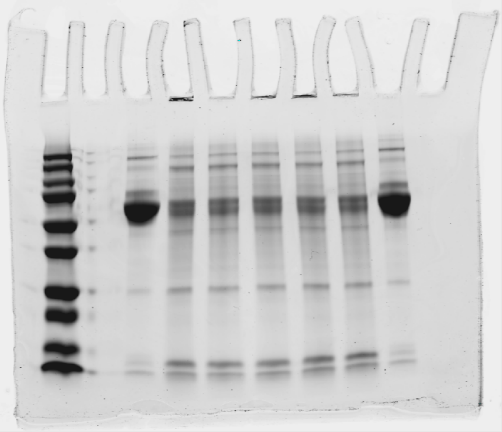


250

75

50

37

25

20

15

10

150

100

***Figure S 1:***  *SDS-PAGE showing the protein corona of PSNPs after 10, 20, 30, 60, 120 min incubation in DMEM^+^.* ***A)*** *50nm (-SM),* ***B)*** *50nm (-CM),* ***C)*** *50nm (-CP), and* ***D)*** *200nm (-CP). The amount of proteins at all-time points for each PSNP was adjusted to load the same amount. The molecular weights of the proteins in the standard ladder are given on the left side of each gel. DMEM^+^ is the medium control.*
